# Comparative Molecular Immunological Activity of Physiological Metal Oxide Nanoparticle and its Anticancer Peptide and RNA Complexes

**DOI:** 10.3390/nano9121670

**Published:** 2019-11-22

**Authors:** Robert K. DeLong, Jeffrey Comer, Elza Neelima Mathew, Majid Jaberi-Douraki

**Affiliations:** 1 Nanotechnology Innovation Center, College of Veterinary Medicine, Kansas State University, Manhattan, KS 66502, USA; jeffcomer@ksu.edu (J.C.); elzamathew@ksu.edu (E.N.M.); 2 Institute for Computational Comparative Medicine and Department of Mathematics, Kansas State University, Manhattan, KS 66502, USA; jaberi@k-state.edu

**Keywords:** nanomaterials, molecular immunology, molecular dynamic simulations, tumor immunology biostatistical analysis

## Abstract

Currently, there is a great interest in nanoparticle-based vaccine delivery. Recent studies suggest that nanoparticles when introduced into the biological milieu are not simply passive carriers but may also contribute immunological activity themselves or of their own accord. For example there is considerable interest in the biomedical applications of one of the physiologically-based inorganic metal oxide nanoparticle, zinc oxide (ZnO). Indeed zinc oxide (ZnO) NP are now recognized as a nanoscale chemotherapeutic or anticancer nanoparticle (ANP) and several recent reports suggest ZnO NP and/or its complexes with drug and RNA induce a potent antitumor response in immuno-competent mouse models. A variety of cell culture studies have shown that ZnO NP can induce cytokines such as IFN-γ, TNF-α, IL-2, and IL-12 which are known to regulate the tumor microenvironment. Much less work has been done on magnesium oxide (MgO), cobalt oxide (Co_3_O_4_), or nickel oxide (NiO); however, despite the fact that these physiologically-based metal oxide NP are reported to functionally load and assemble RNA and protein onto their surface and may thus also be of potential interest as nanovaccine platform. Here we initially compared in vitro immunogenicity of ZnO and Co_3_O_4_ NP and their effects on cancer-associated or tolerogenic cytokines. Based on these data we moved ZnO NP forward to testing in the ex vivo splenocyte assay relative to MgO and NiO NP and these data showed significant difference for flow cytometry sorted population for ZnO-NP, relative to NiO and MgO. These data suggesting both molecular and cellular immunogenic activity, a double-stranded anticancer RNA (ACR), polyinosinic:poly cytidylic acid (poly I:C) known to bind ZnO NP; when ZnO-poly I:C was injected into B16F10-BALB/C tumor significantly induced, IL-2 and IL-12 as shown by Cohen’s d test. LL37 is an anticancer peptide (ACP) currently in clinical trials as an intratumoral immuno-therapeutic agent against metastatic melanoma. LL37 is known to bind poly I:C where it is thought to compete for receptor binding on the surface of some immune cells, metastatic melanoma and lung cells. Molecular dynamic simulations revealed association of LL37 onto ZnO NP confirmed by gel shift assay. Thus using the well-characterized model human lung cancer model cell line (BEAS-2B), poly I:C RNA, LL37 peptide, or LL37-poly I:C complexes were loaded onto ZnO NP and delivered to BEAS-2B lung cells, and the effect on the main cancer regulating cytokine, IL-6 determined by ELISA. Surprisingly ZnO-LL37, but not ZnO-poly I:C or the more novel tricomplex (ZnO-LL37-poly I:C) significantly suppressed IL-6 by >98–99%. These data support the further evaluation of physiological metal oxide compositions, so-called *physiometacomposite* (PMC) materials and their formulation with anticancer peptide (ACP) and/or anticancer RNA (ACR) as a potential new class of immuno-therapeutic against melanoma and potentially lung carcinoma or other cancers.

## 1. Introduction

There is a great deal of current interest in nanoparticle delivery of vaccines and immuno-therapeutics [1,2]. Whereas lipid nanoparticle formulations are in common use, inorganic nanoparticles may act synergistically to induce a beneficial immune response in concert with vaccine antigen or RNA immunogen [3]. For example, physiologically-based zinc oxide (ZnO) nanoparticles (NP) or their composites with iron oxide in complex with a drug and/or RNA with poly inosinic:poly cytdylic acid (poly I:C) generate potent antitumor immunological responses against experimental melanoma in immuno-competent mice [4]. Poly I:C-iron oxide nanoparticle complexes have also been used for immune cell targeting [5]. Certain cytokines such as interleukin-6 (IL-6) are thought to play a key role in cancer progression and resistance to therapy [6]. Whereas others such as IL-1, IL-4, IL-10 may be involved in cancer tolerogenicity [7]. Indeed other cytokines such as IL-2, IL-2, TNF-α, and IFN-γ are thought to regulate the metastatic tumor niche and play a key role in regulating cells of the immune system recruited into this environment [3]. Therefore the effect of inorganic NP such as ZnO and others or their RNA and peptide complexes on tumor associated cytokines is a very important question.

Thus far, much of the work on the immunological activity of ZnO NP has been done in cell culture against a variety of different cell types. Some but not all of these were cells of the immune system or from appropriate tissue compartments where these cells reside such as the skin and spleen. For example treating neutrophils or THP-1 cells with ZnO NP has been shown to affect the expression of a variety of different CD molecules, whereas in PBMC (peripheral blood mononuclear cells) induction of IFN-γ, TNF-α, and IL-12 was reported [8]. That same report referenced an earlier work where ZnO NP-treated RAW264.7 cells (a macrophage line) or bone marrow derived dendritic cells (BMDC) also induced CD and MHC mRNA expression as well as IL-1β and various chemokines (CXCL-5, 9, and 10) [8]. Our lab reported that nanoparticle delivery of tuberculosis antigen 85B induced IL-2 as a bio-marker of antigen presentation in a T cell co-culture assay [9]. We noted molecular and cellular immunogenic activation upon nanoparticle exposure in the ex vivo splenocyte assay [10] or after direct intradermal or intratumoral administration [11,12,13]. Therefore here we endeavored to compare the molecular immunogenicity of zinc, cobalt, magnesium, and nickel oxide NP, finding that the ZnO NP generated superior immunogenicity in these in vitro and ex vivo assays. We then evaluated the in vitro/in vivo immunogenic potential of these NP in complex with an anticancer peptide (ACP) or anticancer RNA (ACR).

## 2. Materials and Methods

### 2.1. Materials

ZnO nanoparticles (NP) and MgO NP were obtained from Sigma-Aldrich (St. Louis, MO, USA) and the nickel oxide NP (NiO) were synthesized as a grey-white powder by our collaborator (Kartik Ghosh, Missouri State University Department of Physics and Materials Science). The purity and elemental composition of all materials were confirmed by elemental analysis and their size was confirmed <100 nm either by dynamic laser light scattering spectroscopy or transmission electron microscopy within the Nanotechnology Innovation Center Kansas State (NICKS, Manhattan, KS, USA). Materials were washed with USP grade 70% ethanol/water followed by pure alcohol, dried in a sterile hood, suspended in sterile phosphate buffered saline (PBS) buffer and sonicated for several minutes in a probe sonicator to disburse the suspension prior to administration to cells or tumor in a 200 microliter volume containing 20 μg/mL NP.

### 2.2. Cytokine Panel

**Figure 1:**
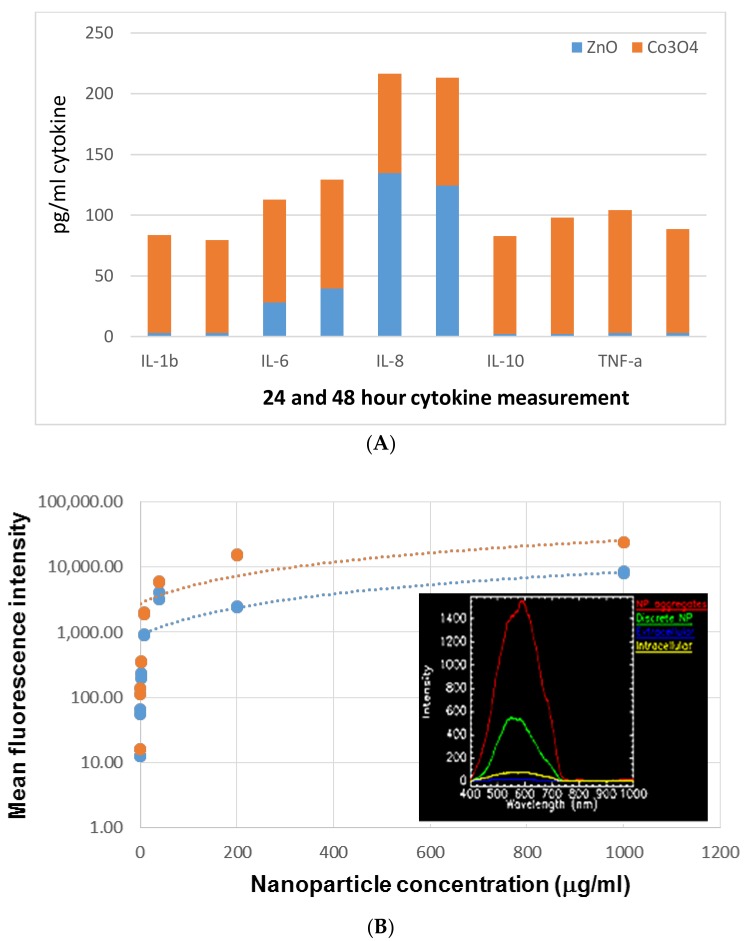
(**A**) Comparing in vitro immunological activity for two physiological metal oxide nanoparticles (NP). (**B**) Dose-response of NP on IL-8 (inset plots HSI results for Co_3_O_4_ NP).

This experiment was conducted similarly to Murray et al. [14]. Briefly, 10^5^ neonatal human epidermal keratinocytes (HEK) were seeded in 96-well plates and treated with nanoparticle concentrations of 1.25, 2.5, 5, 10, 20, 40, 80, 160, and 320 μg/mL for 24 or 48 h. At the end of each time point, the conditioned media was harvested to obtain cytokine levels. Samples were analyzed on the Milliplex analyzer with the detection limit for the analytes IL-1b, IL-6, IL-8, IL10, and TNF-α was between 2.1 to 2.8 pg/mL. The coefficient of variation (CV) of the analysis varied from 0.79% to 40.6% with a median CV of approximately 7–8% depending on the cytokine and NP concentration. The data plot shown in Figure 1B inset were obtained from treating HeLa cells with Co_3_O_4_ NP and imaged assording to manufacturers recommendations using the Cytoviva® nanoscale microscope (Olympus BX51) and hyperspectral imaging (Auburn, AL).

### 2.3. Ex Vivo Splenocyte Assay

**Figure 2:**
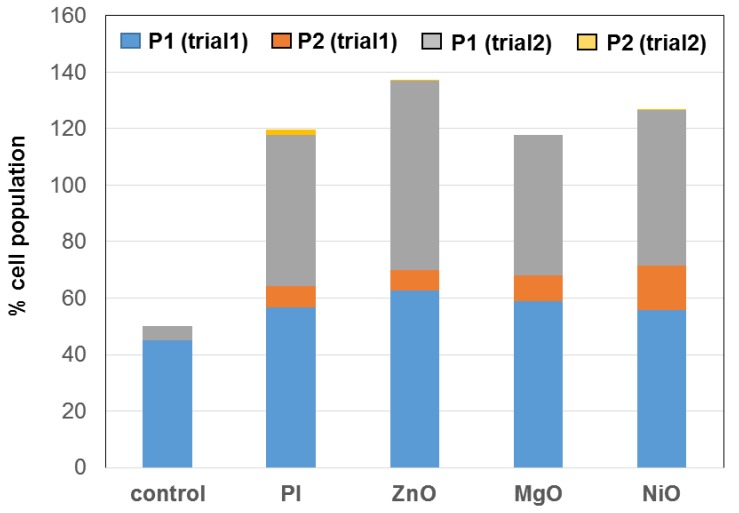
Comparing activity of the physiological metal oxide NP by ex vivo splenocyte assay.

Briefly, this assay was also conducted similarly to what we previously described [10]. Mouse spleens were collected from donor mice from the laboratory of Dr. Sherri Fleming (Kansas State University Biology Department). The spleen was isolated and minced in buffer, the cells were separated from the tissue fragments and incubated in PBS buffer containing a 20 μg/mL suspension of the nanoparticles after which the cells were stained with propidium iodide and analyzed by flow cytometry (BD FACSDiva, Warwick, RI, USA) by the Kansas State University Veterinary Diagnostic Laboratory. The cells separated into two populations in the all event forward scatter (FSC-A) and gated in the P1 and P2 channel (PE-A). The ratio of these two populations from two different trials is plotted in Figure 2.

### 2.4. High Throughput Tumor Proteomics Analysis

BALB/c mice were inoculated with a B16F10 tumor and treated with ZnO-poly I:C complex, as previously described; computational analysis for Table 1 was performed using MATLAB^®^ R2019a. Data for high throughput tumor proteomics analysis were collected in a spreadsheet (Microsoft Excel^®^ 2016, Microsoft Corporation, Redmond, WA, USA) for subsequent calculation using (*x,y*) protein locations. The mean ± SEM (standard error of the mean) were estimated for each set of proteins (probesets). In total, we conducted experiments for unique proteins, approximately 300 (9 × 31) probesets. Each probeset includes a group of six experiments. Pixel intensities at a wavelength of 532 nm, based a on mean and standard deviation analysis, were used to reflect the relative abundance of all proteins in the tumor. Group differences amongst probesets were evaluated with the assumption that data were not normally distributed using Kruskal–Wallis ANOVA on ranks for independent unequal-sized data to perform follow-up multiple comparison tests and identify most significant probesets. P-values for statistical significance were set to ≤0.05.

Effect size calculation: A sample-based effect size is distinguished from the test statistics. For independent samples, effect size from Cohen’s d is determined by calculating the mean difference between your two groups ($\mu_{1}$: control vs $\mu_{2}:$ treated), and then dividing the result by the pooled standard deviation.

$$\mathrm{Effect}\;\mathrm{size}=\frac{\mu_{1}-\mu_{2}}{\mathrm{pooled}\;s}$$

where the pooled standard deviation is obtained by

$$\mathrm{pooled}\;s=\sqrt{\frac{\left(n_{1}-1\right)s_{1}+\left(n_{2}-1\right)s_{2}}{n_{1}+n_{2}-2}\;}$$

We can interpret the effect size measures by the values of Cohen’s d [15,16]; small: 0.2, medium: 0.5, large: 0.80, very large: 1.20, and huge: 2.0. This shows that TNF-α has a medium effect size, IL-2 shows very large effect size, and the other cytokines have huge effect sizes.

### 2.5. Molecular Dynamics

A slab of ZnO was constructed based on the wurzite crystal structure and simulated under the ReaxFF force field [17] using the program LAMMPS [18]. This slab was periodic along the *x* and *y* axes, while the surfaces perpendicular to the *z*-axis, Zn-terminated {0001} facets, were exposed to an aqueous region consisting of 311 water molecules. After equilibration at a temperature of 300 K and pressure of 1 atm, the dimensions of the ZnO slab converged to 20.1 × 17.7 × 8.5 Å^3^. This ZnO structure was replicated 6 × 6 times to produce a continuous slab with dimensions of 120.6 × 106.2 × 8.5 Å^3^, a size suitable for simulations with the LL-37 peptide. For accurate representation of the peptide conformational transitions and better computational efficiency, all further simulations were performed using nonreactive force fields, with the protein described by the CHARMM36m force field [19] and the ZnO described by a CHARMM-compatible force field for ZnO parameterized using experimentally derived adsorption free energies for small molecules on ZnO nanoparticles [20]. The model of the LL-37 peptide was constructed from the NMR structure of the human form [21]. It was initially placed 20 Å from the ZnO surface. Three different initial conditions were created by rotating the peptide 0°, 90°, and 180° around its helical axis, causing a different set of residues to be facing the ZnO surface in the three replicas. The system was filled with water, giving it mean periodic dimensions of 120.6 × 106.2 × 78.7 Å^3^. Na^+^ and Cl^−^ ions were added to obtain an electrically neutral system containing NaCl solution at ≈150 mmol/L. Molecular dynamics simulation was performed using the program NAMD 2.13 [22]. Mass repartitioning of non-water hydrogens enabled the use of a 4 fs timestep [23]. Rigid water molecules were implemented using the SETTLE algorithm [24]. After 50 ps of equilibration in which the atoms of the peptide were restrained to their initial positions, a 500 ns production simulation was performed for each replica. During these simulations, the temperature was maintained at 295 K by a Langevin thermostat and the pressure, at 1.0 atm by a Langevin piston barostat [25].

### 2.6. IL-6 Assay

This assay followed our collaborators’ previously reported conditions except the BEAS-2B cells were cultured and exposed to 10 ug/mL nanoparticles spun down in the presence of 1.3 μg/mL poly I:C and/or LL-37, and IL-6 production relative to LPS or mock infection or medium only controls was assayed by ELISA [26].

### 2.7. MTT Assay

This assay was conducted as we previously described [27]. Briefly 8000 B16F10 cells were added per well of a 96-well plate and treated with 20 mg/mL ZnO-NP loaded in the presence of 3 mM LL37 or carboplatin sedimented from those suspensions and taken up in 10% FBS/DMEM and added to the cells. The cells (*n* = 3 wells/treatment group) were incubated for 96 h and assayed by MTT on a plate reader (Molecular Devices Corp.) at 562 nm absorbance. The plotted bars are the means with standard error shown.

## 3. Results and Discussion

### 3.1. In Vitro Immunogenicity

Our early work suggested particle delivery into the skin can generate significant protective immunity [11,12]. ZnO and Co_3_O_4_ NP have been evaluated against macrophages (RAW264.7) and transformed lung cells (BEAS-2B) [28]. Co_3_O_4_ NP have been shown to penetrate human skin and shown some toxicity and immunostimulatory properties when pulsed with macrophages [29,30]. Thus, here we investigated ZnO and Co_3_O_4_ NP in vitro immunogenicity head-to-head when exposed to a standard human skin cell line, human epidermal keratinocytes (HEK) [14]. In this experiment HEK cells were incubated for 24 and 48 h with NP and their cytokine panels measured by milliplex analyzer (Figure 1).

Figure 1 shows a comparative cytokine panel for an 80 ug/mL dosage of either ZnO or Co_3_O_4_ NP after 24 or 48 h in the left or right bar respectively. With the exception of IL-8, the induction of cancer promoting or tolerogenic cytokines was in the order; Co_3_O_4_ NP > ZnO NP. Dose-response is shown in Figure 1 indicating maximal effect occurs at about 80 ug/mL NP concentration in which case the induction of cytokine begins to top out. Hyperspectral imaging (HSI) experiments confirmed Co_3_O_4_ intracellular uptake (data not shown) and the summary plot of this data is shown in the inset in Figure 1B.

### 3.2. Ex Vivo Splenocyte Assay 

Although iron oxide nanoparticles have been clinically approved and elevated IL-8 levels have been seen when administered systemically, there is some concern reported for their splenic toxicity [31]. The spleen is a rich source and depot of immune cells. Therefore, we investigated ZnO NP in an ex vivo splenocyte assay similar to our earlier report [10]. In this experiment we compared ZnO to nickel oxide (NiO) or magnesium oxide (MgO) NP, where our group and one other have shown that these compositions may be able to functionally load protein or protein-RNA complexes onto their surfaces [32,33,34], making their baseline immunogenicity of great interest as a potential carrier of peptide/protein or RNA-based immuno-therapeutics. Based on earlier dose ranging experiments and our experience, splenocytes were incubated with a 20 μg/mL dose of ZnO, MgO, or NiO NP [9,10,13,31,33]. Splenocytes were isolated from the mouse spleen, exposed to concentrations and stained with propidium iodide (PI) and the cell populations separated and counted by flow cytometry (Figure 2).

As seen in Figure 2 the sorted splenocyte cell populations after PI staining were distinct for ZnO NP in each trial in comparison to untreated or PI treated controls or the other two physiological metal oxide NP tested, MgO or NiO NP. Based on the cellular and molecular immunogenicity of ZnO NP we investigated the immunological activity of loading it with anticancer peptide and RNA described next.

### 3.3. In Vivo Direct Intratumoral Injection Model

**Figure 3:**
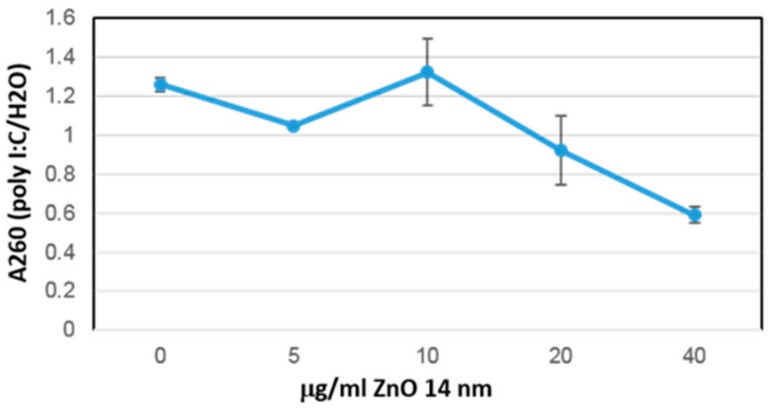
Loss of poly I:C from water supernatant as it associates to ZnO NP after brief spin down in micro-centrifuge.

Poly inosinic:poly cytidylic acid (poly I:C) is an RNA immunogen used widely in cancer research [3,4,5]. In a relatively recent report we reviewed the molecular cell immunology of the metastatic tumor niche and the cytokines which are thought to regulate it [3]. We previously characterized the interaction of poly I:C to ZnO to form poly I:C-ZnO nano-complexes [34] and consistent with another recent study [4], demonstrated inhibition of experimental melanoma in B16F10-BALB/c mice by poly I:C-ZnO nano-complexes relative to ZnO NP or poly I:C controls [13]. Analysis of the poly I:C-ZnO-treated tumors relative to control mice receiving sham PBS intratumoral injection in this immuno-competent B16F10-BALB/c mouse model demonstrates significant antitumor cytokine response (Table 1). In the Figure 3, binding isotherm of poly I:C onto 14 nm zinc oxide (ZnO) nanoparticle measured by the loss in UV absorbance at 260 nm as a function of increased mass per volume (NP) added is shown. This data is in concordance with other measurements in which we have studied the poly I:C nanobio interface to ZnO NP [34]. The table below summarizes cytokines which are thought to regulate immunology of the melanoma metastatic tumor microenvironment and their putative role [3]. Tumors exposed to poly I:C-ZnO had from a 5.8-fold to as much as a 148-fold increase in these antitumor cytokines, the significance of which was demonstrated by Cohen’s d effect size [15,16].

### 3.4. Immunological Activity of ZnO NP Antimicrobial Peptide Complexes

**Figure 4:**
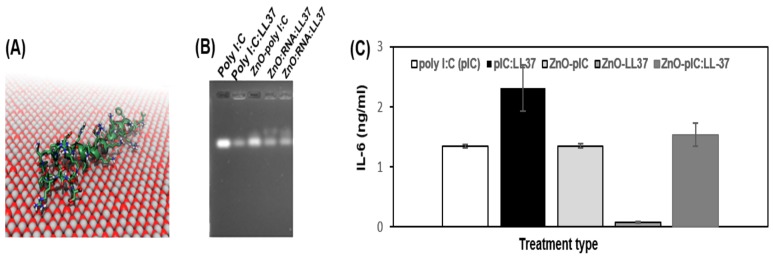
Interaction of LL37 to ZnO NP as shown by molecular dynamic simulation (**A**), electrophoretic gel mobility shift (**B**) suppresses IL-6 cytokine secretion by BEAS-2B transformed lung cells (**C**).

LL-37 is an antimicrobial peptide currently in clinical trials as an intratumoral injection immuno-therapy against melanoma [35]. LL37 binds poly I:C and is thought to partially inhibit its TLR3 based signaling in some cells of the immune system as well as BEAS-2B transformed lung cells [26,36]. Molecular dynamic simulations indicated LL-37 interacts with the surface of ZnO NP and we were able to use its RNA binding properties to assemble RNA-peptide nanoparticle (RNP) onto ZnO NP testing the ability of these novel co-conjugates to inhibit BEAS-2B interleukin-6 (IL-6) expression by ELISA (Figure 4).

As shown in Figure 4A, the molecular dynamics simulations showed a strong interaction between the ZnO surface and the LL-37 peptide. Within 10 ns, the peptide adsorbed to the ZnO in all three simulations. Despite beginning with different orientations of peptide, the final structures in these simulations were remarkably similar. Over the course of 100 ns, the peptides reoriented in such a way that the hydrophobic side chains of the residues L1, L2, F6, I13, F17, I24, F27, L28, L31, and V32 made direct contact with the surface, while most of the charged residues, including D4, K8, K12, R19, D26, and R29, remained extended into the solution. During the remaining 400 ns of simulation, the peptide conformations remained stable, retaining most of their native α-helical structure. A gel shift effect was observed consistent with LL37 binding poly I:C [26] as well as ZnO interacting with both poly I:C or poly I:C-LL37 (3B). Interestingly the delivery of these co-complexes to BEAS-2B transformed lung cells resulted in significant IL-6 suppression by ZnO-LL37 and not the RNA or RNA-peptide complex (3C).

### 3.5. Combination Therapy Experiment

**Figure 5:**
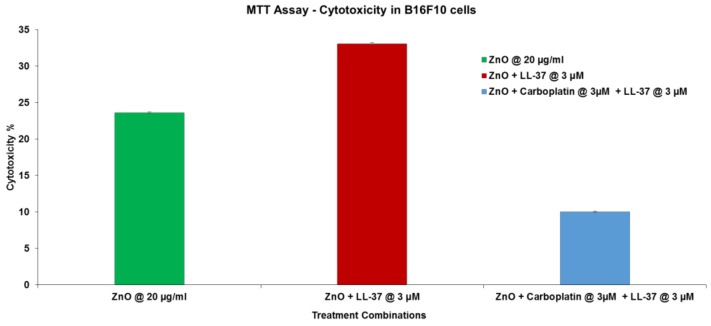
Cytotoxic activity of ZnO, ZnO-LL37 or ZnO-LL37/carboplatin combination.

As shown in Figure 5 next we investigated whether loading ZnO-LL37 with chemotherapy drug would improve cytotoxicity to B16F10 melanoma cells (Figure 5).

As shown in Figure 5, loading the ZnO-LL37 complex with melanoma-specific chemotherapy drug did not show any additional advantage. In this case ZnO NP gave 20% cytotoxicity, the ZnO-LL37 increase cytotoxicity to 30%, but at an equivalent dose (3 μM) and exposure (96 h) we observed no advantage of the additional drug.

## 4. Conclusions

Overall these data suggest that the physiological metal oxide NP are likely to be quite variable in their immunological activity dependent on the composition and the protein and RNA to which they are complexed. This can be seen as shown in Figure 1 at a dosage of 80 μg/mL in the differential effects of ZnO or Co_3_O_4_ NP on IL-8 considered an immuno-toxicity marker versus IL-6 considered a pro-cancer cytokine associated with drug resistance [3,6]. ZnO activity was further distinguished in the ex vivo splenocyte assay [10] where relative to MgO or NiO NP differences in the two distinct propidium iodide populations were shown, these data suggesting distinct activity of these metal oxide NP on splenocytes as well. Complexation of ZnO to immunogenic RNA (poly I:C) or anticancer peptide (ACP) such as LL37 also had quite distinct effects, in the former case inducing cytokines associated with regulating the metastatic tumor niche (Table 1) and in the latter inhibiting pro-tumor cytokine (Figure 4). These data suggest that the anticancer immunity of either ZnO-RNA or ZnO-peptide will likely need to be evaluated on a case by case basis and may vary depending on the cell type, tissue and tumor to which they are administered. A recent report suggests that loading liposome with butyric acid may lower IL-8, IL-6, TNF-α, and TGF-β similar to our data [37]. Combination therapies show great promise such as the recent report by Serati et al. who used nanoparticles loaded with quercetin and gemcitabine and surface-decorated with hyaluronic acid (HA) which improved anticancer activity and lowered their interleukin profile [38]. An analogous LL37/carboplatin co-loaded ZnO NP formulation did not show combination or synergistic effects. This suggests future work on surface decoration of ZnO NP may warrant further investigation.

## Figures and Tables

**Table 1:** Intratumoral administration of ZnO-poly I:C nanocomplex induces tumor-regulating cytokine response relative to PBS sham injected controls.

Cytokine	Putative Role in Metastatic Tumor Niche	Effect of ZnO-Poly I:C (Fold Over Control)	Effect Size Analysis (Cohen’s d Measure)
**TNF-α**	Chemotaxis, leukocyte recruitment, extracellular killing	148.3+/−33.7	−0.59
**IFN-γ**	Secreted by cancer, T, natural killer (NK) cells and macrophages (Macs), MHCI/II, involved in Th1 and Th2	6.0+/−0.92	−2.81
**IL-2**	CD8 and NK activation, activates antigen presentation and B cell response (BCR)	5.8+/−3.2	1.24
**IL-12**	Growth factor, increases NK action, stimulates antibody production	9.9+/−4.3	18.58
